# Antipsychotic-associated psoriatic rash – a case report

**DOI:** 10.1186/s12888-017-1411-2

**Published:** 2017-07-04

**Authors:** Camelia-Eugenia Bujor, Torkel Vang, Jimmi Nielsen, Ole Schjerning

**Affiliations:** 1 Department of Psychiatry, Aalborg University Hospital, Aalborg, Denmark; 2 Department of Clinical Medicine, Aalborg University, Aalborg, Denmark; 3 Mental Health Centre Glostrup, University of Copenhagen, Copenhagen, Denmark

**Keywords:** Aripiprazole, Quetiapine, Psoriasis, Side effects, Schizophrenia, Antipsychotic drugs, T cells, Cytokines

## Abstract

**Background:**

Antipsychotics are a heterogeneous group of drugs. Although, antipsychotics have been used for years, unexpected side effects may still occur. With this case report we focus on a possible association between psoriasis and antipsychotics.

Data on the patient’s course of psychiatric disease, onset of psoriasis and its evolution were extracted from the patient’s medical files.

**Case presentation:**

We present a case of a 21-year-old female diagnosed with schizophrenia. She was initially treated with quetiapine, and later switched to aripiprazole due to weight gain. After initiation of antipsychotic treatment, the patient suffered from severe psoriasis lesions.

**Conclusions:**

Antipsychotics may possess immunological properties that may be involved in immune-mediated conditions, such as psoriatic rash. Further studies are warranted to determine causality and mechanism.

## Background

Psoriasis, which affects up to 3% of the adult population, is a chronic inflammatory skin disease characterized by excessive growth and aberrant differentiation of keratinocytes. The etiology of psoriasis is incompletely understood but involves both genetic risk factors and environmental triggers. Genetic analyses have implicated genes associated with development/differentiation/function of immune cells but also genes important for epidermal differentiation and skin barrier function [1]. The recent introduction of biological agents as a treatment option for psoriasis has enhanced our knowledge of the pathogenesis of the disease. Interestingly, interleukin 12 (IL-12) / IL-23 antagonists have been demonstrated to be efficient anti-psoriatic drugs for patients with moderate to severe disease burden [2]. This suggests a role for T helper cells type 1 (Th1 cells, characterized by production of interferon γ and tumor necrosis factor α) and/or T helper cells type 17 (Th17 cells, characterized by secretion of IL-17A/F and IL-22) in the pathogenesis of psoriasis as the differentiation/stability of these cells are stimulated by IL-12 and IL-23, respectively. However, recent studies have indicated that both IL-23 antagonists and IL-17 antagonists are efficient anti-psoriatic drugs, suggesting that abnormalities in the IL-23/Th17 axis are of special importance in the pathogenesis of psoriasis [2].

Several factors are known to exacerbate psoriasis. These include traumatic injury to the skin, physical and psychological stress, cold weather, and excessive alcohol intake. Administration or withdrawal of certain drugs may also trigger psoriasis or exacerbate existing psoriasis. Especially, lithium, beta-blocking agents, carbamazepine, and sodium valproate have been associated with triggering or worsening of psoriatic rash [3]. Interestingly, all these drugs show immunomodulatory effects and as psoriasis clearly is an immune-mediated disease a common mechanism is most likely.

Antipsychotics, which remain the cornerstone in the treatment of schizophrenia and other psychoses, affect several organ systems beyond the central nervous system. Along these lines, immunomodulatory effects of antipsychotics have also been described [4]. With these effects in mind and with the aim to increase focus on this serious adverse effect, we present a case report with a patient developing psoriatic rash during antipsychotic treatment with quetiapine and aripiprazole.

## Case presentation

The patient is now a 21-year-old woman that was diagnosed with ICD-10 F20.0 paranoid schizophrenia at the age of 17. All psychiatric charts were reviewed to establish a comprehensive sequence of relevant medical events. The patient’s general practitioner was contacted and all appointments regarding skin conditions were evaluated. The treating dermatologist was contacted as well and charts were reviewed.

The Patient have no diagnostically confirmed family history of psoriasis. At the age of two the patient had been seen twice by the general practitioner (GP) due to eczema on the eyelids and in the palm of her hands. At the age of five she had another contact with her GP concerning birthmarks and a plantar wart.

**Fig. 1:**
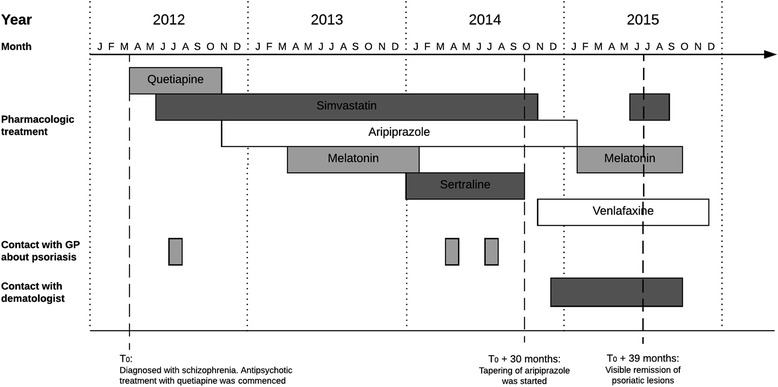
Graphical presentation of medication history and medical events

The medication history and medical events are summarized in Fig. 1. At the age of 17 she was admitted to a psychiatric children’s ward for 3 months and diagnosed with schizophrenia. Thereafter, she was followed in a psychiatric outpatient clinic. In the following, the time of the diagnosis is referred to as T_0_. Antipsychotic treatment with quetiapine was initiated at T_0_. Routine blood monitoring was performed during treatment start-up and at regular basis afterwards. At T_0_ plus 2 months treatment with simvastatin was initiated due to dyslipidemia with elevated blood-cholesterol. In the following months the patient experienced unacceptable weight gain. According to the patient, she experienced the first psoriatic lesions about 2 months after the initiation of quetiapine. First contact with the GP concerning possible psoriasis was at T_0_ plus 4 months with an approximately 2 cm wide skin lesions on the left arm as well as lesions on both elbows and knees. Topical treatment with various agents including Daivobet gel, Locoid cream, and Xamiol gel was commenced by the GP but the psoriasis lesions worsened. At T_0_ plus 7 months, treatment with quetiapine was discontinued and aripiprazole was initiated at 5 mg/d and gradually increased to 15 mg/d within 2 months. At T_0_ plus 12 months treatment with melatonin was initiated due to insomnia. Melatonin was discontinued at T_0_ plus 20 months. At the same time, treatment with sertraline was initiated; the dosage was increased slowly up to 200 mg/d due to anxiety and mild to moderate depressive symptoms. Concomitantly, the dosage of aripiprazole was increased to 25 mg/d. The patient had another contact with her GP, at T_0_ plus 25 and 28 months, concerning her psoriasis. At T_0_ plus 30 months the patient wished to discontinue all her pharmacologic treatment due to possible side effects of the treatment. She had experienced emesis and vomiting for several months and had been evaluated by a gastroenterologist without finding any satisfactory physical explanation for the symptoms. Her treating psychiatrist suspected that the gastric symptoms were caused by simvastatin, which was discontinued at T_0_ plus 30 months. However, the patient was convinced that sertraline and aripiprazole worsened her anxiety symptoms and psoriatic lesions. Therefore, sertraline was discontinued by the patient at T_0_ plus 30 months. The patient wished to discontinue aripiprazole as well, but due to the risk of psychotic relapse she agreed to slowly tapering of the drug, which was discontinued at T_0_ plus 35 months. Due to ongoing anxiety and depressive symptoms treatment with venlafaxine was initiated at T_0_ plus 31 months. Due to the extensive worsening and lack of treatment results the patient was referred to a dermatologist at T_0_ plus 32 months. Treatment with intensive phototherapy (UVB and Bucky rays) for 3 months gave only minor improvement, even when two more ointments (Diprosalic, Propiosalic) were added. Treatment with melatonin was resumed at T_0_ plus 34 moths and simvastatin was resumed at T_0_ plus 38 months.

At T_0_ plus 39 months, the psoriatic lesions had almost entirely disappeared without any modification of the previously commenced dermatological treatment. The recommenced treatment with simvastatin and melatonin did not worsen the psoriatic lesions in the following period. So far, the patient’s psychiatric state has only been slightly affected by the discontinuation of aripiprazole (mild worsening of hallucinations). All blood tests were normal throughout the course of events.

## Discussion

The most common agents involved in drug-associated psoriasis are beta-blockers, lithium, anti-malarials, anti-convulsants, and NSAIDs. Apart from olanzapine, there is a paucity of reports suggesting a possible link between the use of antipsychotics and psoriasis. To our knowledge, the current report is the first to describe a potential association between treatment with two other antipsychotics (quetiapine and aripiprazole) and psoriasis. However, a causal relationship cannot be established at this time since the pharmacological treatment of the patient was rather complex with several different drugs administered during the covered treatment period. Additional factors (both environmental and intrinsic to the patient) may also have affected the clinical course of the skin disease.

The exact mechanisms for drug-associated psoriasis are not known and most likely vary between different drugs. However, there seems to be some common features. These include a direct effect of the drug on keratinocytes as well as immunomodulatory properties of the drug. This also fits with the current view of psoriasis as a disease resulting from a complex interplay between environmental factors, keratinocytes, and components of both the innate and the adaptive immune system [5]. Beta-blockers represent a good example in this regard. Beta-adrenergic stimulation results in increased intracellular levels of cAMP in both keratinocytes and immune cells, leading to subsequent inhibition of cellular proliferation and for immune cells also reduced cellular activation [6–10]. With beta-blocking agents the opposite responses may occur; enhanced proliferation of keratinocytes and immune cells and augmented immune responses (i.e. a relative loss of immunological tolerance) [6, 11, 12]. Likewise, lithium may stimulate keratinocyte proliferation, presumably through inhibition of glycogen synthase kinase 3 (GSK3), and also exerts immune-regulatory functions [13, 14]. Moreover, all the above-listed drugs associated with psoriasis have been demonstrated to affect immune cells in various ways.

Several different drugs were prescribed to our patient, and they could all potentially have contributed to the subsequent development of psoriasis. To get a more objective evaluation, the Naranjo ADR scale [15] was applied for each drug administered. The score for quetiapine was 4 and, for simvastatin 3, for aripiprazole 2 and sertraline 2. These scores suggest a “possible” association between the event (psoriasis) and the treatment. The scores for venlafaxine and melatonin were negligible (0 and −1, respectively). A broader discussion on the role of quetiapine and aripiprazole is given below. Simvastatin as a contributing factor for the patient’s psoriasis is less likely since the drug was discontinued and later reintroduced without affecting the clinical course of the skin disease. Furthermore, preclinical data indicate that statins favor stability of regulatory T cells (also known as Tregs, which inhibit conventional T cells) over conventional T cells. The net result may be increased immunological tolerance. In agreement with this, statins have been proposed as a potential treatment option in psoriasis and other immune-mediated diseases [16–18]. However, here it should also be mentioned that there are anecdotal reports linking certain statins (e.g. atorvarstatin) to psoriasis [19]. Regarding sertraline, it is an antidepressant drug belonging to the group of SSRIs (selective serotonin reuptake inhibitors), which are known to exert anti-inflammatory actions [20]. Sertraline has also been demonstrated to represent effective treatment in an animal model of rheumatoid arthritis [21]. Based on this, a link between sertraline and psoriasis seems less likely. Besides, there are no reports suggesting such a connection.

As already mentioned, the Naranjo ADR scores for quetiapine and aripiprazole indicated a “possible” association for a drug-induced event. The timing of the antipsychotics given to the patient (introduction of quetiapine at T_0_ and switch to aripiprazole at T_0_ plus 7 months) combined with the clinical course of the psoriasis (debut at T_0_ plus 4, and remission at T_0_ plus 39 months) make it difficult to assess the individual roles of the two antipsychotics in the patient’s skin disease. One possibility is that there is a class-effect, i.e. antipsychotics in general enhance the risk of psoriasis in susceptible patients.

We searched pubmed and embase for published case reports and case series implying an association between antipsychotics and development or worsening of psoriatic rash. We identified only two case reports [22, 23], covering a total of three patients. Summary of these case reports are shown in Table 1. All these patients experienced exacerbation of the skin disease while treated with olanzapine. Not all cases might have been identified in our search. Language was restricted to English, German, Dutch and Scandinavian language. Some review articles refer to cases concerning exacerbation of psoriasis during treatment with risperidone and quetiapine [24]. However, these cases were not published in journals indexed for medline or pubmed. These case reports should therefore be interpreted within their limitations. As the association between antipsychotics and psoriasis is not well established, psychiatrists may not have been aware of this potential association and a substantial underreporting may have occurred. In addition, we used search terms including only case reports indexed this way and not including patients from clinical studies.

**Table 1:** Characteristics of published cases associating psoriasis and antipsychotic treatment

Author [Reference]	No. of cases	Sex	Age	Psychiatric diagnosis	Antipsychotic treatment	Diagnostic assessment of skin condition	Possible effect of antipsychotic treatment	Comments
Ascari-Raccagni et al. [22]	1	Female	52	Schizophrenia (subtype not specified)	Olanzapine	Dermatologist	Exacerbation of chronic large plague psoriasis	Dramatic worsening of psoriasis two weeks after initiation of olanzapine treatment. Cyclosporin treatment was initiated. Following course of disease is not described.
Latini et al. [23]	2	male	38	Psychosis with schizophrenic symptoms	Olanzapine	Unknown	Exacerbation of a pre-existing mild psoriasis	Known large plaque psoriasis from the age of 14. Worsening of psoriatic lesions when olanzapine treatment was initiated. Lesions bettered when olanzapine was discontinued and cyclosporine treatment was initiated. Lesions reappeared when olanzapine was reinitiated 6 months later.
		male	28	Psychosis	Olanzapine	Psychiatrist	Triggering of psoriasis in a patient with familial history of psoriasis	Family history of psoriasis but no clinical manifestations of psoriasis before initiation of olanzapine treatment. Lesions appeared 20 days after initiation of olanzapine. Lesions cleared one month after discontinuation of olanzapine. Lesions was treated with topical treatment with salicylic acid and clobetasol propionate.

Given dopamine’s ability to bind to various dopamine receptors and thereby modulate cAMP levels in both keratinocytes and immune cells [25, 26], one hypothesis may be that antipsychotics through dopamine blockade can affect keratinocytes and/or immune cells, ultimately leading to psoriasis in predisposed individuals. The term “predisposed individuals” is important here since a case-control study using diagnostic and treatment data from general practice in Britain found reduced risk of psoriasis associated with the use of atypical antipsychotics, mainly accounted for by olanzapine [27]. In comparison, the same study clearly showed enhanced risk for psoriasis associated with lithium. Evidently, additional studies are required to clarify this issue. The typical debut of psoriasis is between 20 and 40 years of age. In the case described in this paper psoriasis may have developed coincidentally with the prescription of antipsychotic drugs or any other agents. Furthermore, psoriasis is generally considered to be a chronic disease where significant fluctuations of symptom severity can be seen over time, and the natural course of the disease may be rather unpredictable [28]. Therefore, the described clinical improvement experienced by the patient may have occurred independently of the drugs administered. There are also well-known triggering factors for psoriasis, e.g. infections and stress. Infections as a triggering factor in our patient were excluded on the basis of normal blood tests. Regarding stress, the patient’s psychiatric condition got worse when she moved to another apartment located in an extremely noisy area; she experienced sleep disturbances as well as augmented paranoid delusions and high level of anxiety. This psychotic episode happened during treatment with aripiprazole and may also have affected the course of the skin disease.

Some studies have found increased risk of psychiatric disorders like anxiety and depression among patients with psoriasis [29–31]. Conversely, psoriasis is more prevalent among patients with schizophrenia [32]. The reason for the latter is not known, but it is tempting to speculate that the immune activation seen in connection with psychosis may play a role. In agreement with this, a recent study demonstrated constitutively enhanced levels of IL-23 in patients with schizophrenia [33]. As already mentioned, IL-23 plays an important role in the pathogenesis of psoriasis. Furthermore, both neuronal tissue and skin tissue are ectodermally derived and may share similar pathogenic pathways. Miyaoka et al. presented three cases where schizophrenia was associated with psoriasis [34]. Interestingly, they found that the exacerbation and remission of the skin manifestations of psoriasis closely correlated with the psychosis [34].

## Conclusions

In summary, we report a possible association between treatment with antipsychotic drugs (quetiapine and aripiprazole) and psoriasis. Evidently, more studies are required to clarify if there is a causal relation and whether there is a class effect linked to antipsychotic drugs in general.

## Abbreviations

ADR: Adverse drug reaction
GP: General practitioner
GSK3: Glycogen synthase kinase 3
IL: Interleukin
SSRI: Selective serotonin reuptake inhibitor
T: Time
Th: T helper cells
Tregs: Regulatory T cells
